# Evaluating the effect of *TLR4*-overexpressing on the transcriptome profile in ovine peripheral blood mononuclear cells

**DOI:** 10.1186/s40709-020-00124-3

**Published:** 2020-07-29

**Authors:** Xiaofei Guo, Jinlong Zhang, Yao Li, Jing Yang, Yihai Li, Chunxiao Dong, Guoshi Liu, Zhengxing Lian, Xiaosheng Zhang

**Affiliations:** 1 Tianjin Institute of Animal Husbandry and Veterinary Medicine, Tianjin Academy of Agricultural Sciences, Tianjin, 300381 China; 2 College of Animal Science and Technology, China Agricultural University, Beijing, 100193 China

**Keywords:** *TLR4*-overexpressing, Peripheral blood mononuclear cells, Transcriptome, Sheep

## Abstract

**Background:**

Toll-like receptor 4 (TLR4) plays an important role in the elimination of Gram-negative bacteria infections and the initiation of antiinflammatory response. Using the technology of pronuclear microinjection, genetically modified (GM) sheep with *TLR4* overexpression were generated. Previous studies have shown that these GM sheep exhibited a higher inflammatory response to Gram-negative bacteria infection than wild type (WT) sheep. In order to evaluate the gene expression of GM sheep and study the co-expressed and downstream genes for *TLR4*, peripheral blood mononuclear cells (PBMC) from *TLR4*-overexpressing (Tg) and wild type (WT) sheep were selected to discover the transcriptomic differences using RNA-Seq.

**Result:**

An average of 18,754 and 19,530 known genes were identified in the Tg and WT libraries, respectively. A total of 338 known genes and 85 novel transcripts were found to be differentially expressed in the two libraries (*p* < 0.01). A differentially expressed genes (DEGs) enrichment analysis showed that the GO terms of inflammatory response, cell recognition, etc. were significantly (FDR < 0.05) enriched. Furthermore, the above DEGs were significantly (FDR < 0.05) enriched in the sole KEGG pathway of the Phagosome. Real-time PCR showed the *OLR1*, *TLR4* and *CD14* genes to be differentially expressed in the two groups, which validated the DEGs data.

**Conclusions:**

The RNA-Seq results revealed that the overexpressed *TLR4* in our experiment strengthened the ovine innate immune response by increasing the phagocytosis in PBMC.

## Background

Most Gram-negative bacteria are considered to be harmful to their hosts, many of them (such as *Brucella melitensis*, *Salmonella enterica* and *Escherichia coli*) are pathogenic to both animals and humans. Due to the long-term exposure to pathogens, the mammalian innate immune system has evolved a faithful mechanism to rapidly sense and respond to Pathogen-Associated Molecular Patterns (PAMPs) based on their pattern recognition receptors (PRRs) [1]. As a member of PRRs, Toll-like receptor 4 (TLR4) plays an important role in Gram-negative bacterial infection elimination and inflammatory response initiation [2]. Lipopolysaccharide (LPS), which is the principal component of the outer membrane of Gram-negative bacteria, can be recognized by TLR4 in the mammalian innate immune system [3–5]. Subsequently, myeloid differentiation factor 88 (MyD88), interleukin (IL)-1 receptor-associated kinase (IRAK), and tumor necrosis factor (TNF) receptor-associated factor 6 (TRAF6) are recruited to the intracellular portion of TLR4 leading to the activation of multiple intracellular cascades, including extracellular signal-regulated kinases (ERKs), c-Jun N-terminal kinases (JNKs), and nuclear factor kappa B (NF-κB) [6]. Finally, pro-inflammatory cytokines are induced to fight the Gram-negative bacteria infection.

Given the importance of TLR4 for Gram-negative bacteria recognition and elimination, strengthening its efficacy in livestock seems necessary for the prevention of zoonotic diseases. Therefore, using the technology of pronuclear microinjection, GM sheep with *TLR4* overexpression were generated [7–9]. Changes in the inflammatory responses, Mitogen-activated Protein Kinase Signalling, oxidative stress response, etc. for the above GM sheep were studied, revealing that the sheep with *TLR4* overexpression exhibited a more sensitive response to Gram-negative bacteria. In our previous studies, under the stimulation of LPS, the phagocytosis index of the monocytes in transgenic sheep was higher than that of non-transgenic sheep in vitro, and tissue sections showed that transgenic individuals launched an inflammation response more quickly in vivo [7]. Also, the monocytes of transgenic sheep could phagocytize *Escherichia coli* with higher adhesive capacity [9]. Our further findings for *Salmonella enterica* infection indicated that the overexpression of TLR4 enhances phagocytosis through PI3K signalling, as well as the subsequent activation of actin polymerization and scavenger receptors in sheep monocytes/macrophages [10].

However, GM sheep with pronuclear microinjection were susceptible to some risks of uncertainty in gene integration sites [11]. Due to the random nature of transgene integration, several potential problems have emerged; for example, exogenous genes may be poorly or inappropriately expressed, leading to some endogenous genes being disrupted or having disordered expression (even activating oncogenes) [12]. In the present study, to evaluate the safety of GM sheep at the gene expression level and study the co-expressed and downstream genes for *TLR4*, *TLR4*-overexpressing and wild type sheep were chosen to determine the transcriptome differences in peripheral blood mononuclear cells.

## Methods

### Construction of the transgenic vector for *TLR4*

Ovine spleens were collected and frozen in liquid nitrogen instantly for RNA extraction. Then, cDNA sequence was amplified using reverse transcript-PCR (extension for 15 min at 37 °C, and termination for 5 s at 85 °C). The *TLR4* cDNA sequence was amplified based on the sequence AM981302 (GenBank Accession No, NCBI). Two restriction sites of *EcoR*I and *Sma*I were added to the primers (Forward: ccg gaa ttc ATG GCG CGT GCC CGC CG; reverse: tcc ccc ggg gGG TGG AGG TGG TCG CTT CTT GC) for cloning into the vector. The *TLR4* PCR steps were as follows: initial denaturation at 98 °C for 5 min, followed by 35 cycles of 20 s at 98 °C, 20 s at 65 °C, 2 min at 72 °C, and a final extension step for 10 min at 72 °C, then stored in 4 °C. After double enzyme digestion of *EcoR*I and *Sma*I (NEB, Beverly, MA, USA), the PCR products of *TLR4* were connected to the p3S-LoxP vector, and the *TLR4* expression vector was generated [7, 13].

### Multiple ovulation and embryo transfer for *TLR4*-overexpressing sheep production

The procedure for multiple ovulation and embryo transfer (MOET) was performed in Dorper (donor) and Hu sheep (recipient). During the spring season, controlled internal drug release device (CIDR, Pharmacia and Upjohn, New Zealand) was inserted into all the experimental ewes’ vaginas for 14 days. Meanwhile, 5 mL of vitamin AD was intramuscularly injected to protect the vaginal epithelium. Intramuscular injections of FSH (Ningbo, Zhejiang, China) were given to donors at 12 h intervals for a 4-day period, which extended from 2.5 days before CIDR removal to 1 day after CIDR removal. Then, a total of 0.1 mg cloprostenol (Ningbo, Zhejiang, China) was intramuscularly injected at the time of seventh FSH injection. Finally, for ovulation induction, a dose of 100 IU LH (Ningbo, Zhejiang, China) was intramuscularly injected 48 h after CIDR removal. Laparoscopic insemination with Dorper’s semen was performed 8 h later. The time of CIDR removal for the recipients was 10 h later than that for the donors. Then, 330 IU PMSG (Ningbo, Zhejiang, China) and 15 μg Luteinising Hormone Releasing Hormone A3 (Ningbo, Zhejiang, China) were separately intramuscularly injected into the recipients during CIDR removal and 36 h after CIDR removal.

Seventy-two hours after CIDR removal, all donors were subjected to a surgical operation (both oviducts flushed with sterile phosphate-buffered saline) to collect the fertilized eggs. The *TLR4* expression vector was microinjected into a total of 402 zygotes in vitro, and 2 to 5 microinjected zygotes were transferred into the recipient’s oviduct ipsilateral to the well ovulated ovary within 1 h. 355 surviving microinjected zygotes were transferred into 103 recipients. Forage and drinking water were provided ad libitum during the duration of the experiment.

### Screening of the *TLR4*-overexpressing sheep and sample collection

Southern blot was used to identify the *TLR4*-overexpressing sheep. The probe for Southern blot was 671 bp, which was amplified through the following primers: P-F, 5΄-ACTGGTAAAGAACTTGGAGGAGG-3΄, and P-R, 5΄-CCTTCACAGCATTCAACAGACC-3΄. The PCR proceeded as follows: initial denaturation for 5 min at 95 °C, followed by 30 cycles of denaturation for 30 s at 95 °C, annealing for 30 s at 72 °C, and extension for 30 s at 72 °C, with a final extension for 10 min at 72 °C, followed by storage at 4 °C. The probe was purified by a DNA Purification Kit (TIANGEN Biotech Co., Ltd, Beijing, China) and stored at − 20 °C for the following experiment. Genomic DNA from the ear tissue of 2 week-old lambs was digested with *Hind*III (NEB, Beverly, MA, USA), and the enzyme-digested products were subjected to hybridization of the probe labeled with digoxigenin (Roche Diagnostics, Mannheim, Germany). The expected size of the exogenous *TLR4* fragment was 2771 bp, and the endogenous *TLR4* fragment was 5118 bp. Twenty-seven descendants were successfully born, under analysis of southern blot, 3 male and 4 female were found to be positive for the presence of exogenous *TLR4* CDS.

When the three *TLR4*-overexpressing male grown up, they were selected as the Tg (Transgenic) group; and three adult ram of wild type without microinjection were randomly selected as the WT (wild type) group. All the experimental sheep in Tg and WT groups were 3-year old, they were raised in the same rearing environment and fed the same forage and commercial concentrate supplement 6-month-round. All of the six experimental sheep were not related to each other. Peripheral blood was aspirated from their jugular vein in the spring, and peripheral blood mononuclear cells (PBMCs) were isolated with a sheep lymphocyte separation medium (TBD, Tianjin, China). PBMCs were lysed in Trizol (Invitrogen, Carlsbad, USA) for total RNA extraction, and then stored at − 80 °C for RNA sequencing.

### Library construction and sequencing

Based on the manual of the NEBNext^®^ UltraTM RNA Library Prep Kit for Illumina (NEB, Ipswich, MA, USA), each RNA sample of 3 μg was purified using poly-T oligo-attached magnetic beads. Then, fragmentation was performed using divalent cations at an elevated temperature in NEBNext First Strand Synthesis Reaction Buffer (5X) and a random hexamer primer and M-MuLV Reverse Transcriptase was used to synthesize the first strand cDNA. The second strand cDNA was subsequently synthesized using DNA Polymerase I and RNase H. The fragments were purified by an AMPure XP system (Beckman Coulter, Beverly, MA, USA) and amplified by Universal PCR primers and an Index (X) Primer to acquire a cDNA library of 250–300 bp in length. After assessment using an Agilent Bioanalyzer 2100 system (Agilent Technologies, Santa Clara, CA, USA), qualified libraries were sequenced on the Illumina Hiseq platform (Illumina, San Diego, CA, USA) and 150 bp paired-end reads were generated.

### Reads filtering and differential expression analysis

In accordance with the requirement of bioinformatics analysis, clean reads were obtained by removing contaminated reads (those containing the adapter, those containing poly-N and low quality reads) from the raw reads. Then bases whose Qphred = − 10log_10_ (sequencing error rate) with values greater than 20 or 30 as a percentage of total bases (also known as Q20 or Q30), and GC content of the clean data was calculated. All the downstream analyses were based on the above clean data with high quality. Clean reads were mapped to the sheep genome (https://www.ncbi.nlm.nih.gov/genome/83?genome_assembly_id=351950) using Hisat2 v2.0.5 (https://daehwankimlab.github.io/hisat2/) [14]. The reads numbers mapped to each gene were counted by feature Counts v1.5.0-p3 (https://sourceforge.net/projects/subread/files/subread-1.5.0-p3/) [15]. Novel transcripts which could not be annotated into the genome database were assembled using StringTie software (https://ccb.jhu.edu/software/stringtie/) [14], and these novel transcripts were annotated and predicted with Pfam database (https://pfam.xfam.org/) [16]. After calculating the expression values (read counts) of all genes in each sample, the correlation coefficient between the intra- and inter-group samples were calculated, and a heat map was drawn to visually show the differences in the expression patterns between the samples and the repeats of the samples within the group. Principal component analysis (PCA) was performed on the samples correlation matrix. Differential expression analysis of the two groups was performed using the DESeq2 R package (1.12.3) (https://www.rdocumentation.org/packages/DESeq2/versions/1.12.3) [17].

### Enrichment analysis of GO and KEGG

Differentially expressed genes between Tg and WT were identified prior to the Gene Ontology (GO) and Kyoto Encyclopedia of Genes and Genomes (KEGG) enrichment analyses using the WebGestalt 2019 based on the data of *Bos taurus* (http://www.webgestalt.org/option.php) [18]. A GO term or pathway with corrected *p* value (*p*_adj_) ≤ 0.05 was defined as a significantly enriched in differentially expressed genes [19, 20].

### Analysis of genes associated with the expression pattern of *TLR4* in Tg sheep

Pearson correlation analyses of genes expression were performed on all of the detected genes. A network containing known genes, which expression pattern associated with *TLR4,* was built using Cytoscape v3.5.0 (https://cytoscape.org/) [21]. Based on the human database in String (https://string-db.org/), an interaction network containing proteins whose gene expression pattern associated with *TLR4* was also generated.

### Real-time PCR of internalization-associated genes

According to the manufacturer’s instructions for the Prime Script^®^ RT reagent Kit (Takara Bio Inc., Dalian, China), the remaining RNA (also extracted from the six adult rams) was subjected to reverse-transcription PCR for cDNA synthesis. After incubation at 37 °C for 15 min and deactivation at 85 °C for 5 s, we obtain the template for real-time PCR. All of the cDNA from six samples are mixed, and a standard sample is formed. A SYBR^®^ Premix Ex Taq™ kit (Takara Bio Inc., Dalian, China) was used in the LightCycler^®^ 480 Real-Time PCR system (Roche Applied Science, Mannheim, Germany) to quantify the expression levels of *NFKB1*, *CD14*, *MYD88*, *TLR4*, and *ICAM*-*1* in two groups of microinjection descendant (Table 1). The reaction system of RT-PCR (20 μL) contained 10 μL SYBR^®^ Premix Ex Taq™ II, 2 μL cDNA, and 0.8 μL of forward and reverse primers, respectively, with the rest of the volume supplemented by ddH_2_O. The program for the real-time PCR reaction was as follows: denaturation at 95 °C for 3 min, followed by 40 cycles of 10 s at 95 °C and 10 s at 60 °C. Then, the melting curves were collected. All reactions were performed in triplicate. β-actin was used as the housekeeping gene, and the method of 2^−ΔΔCT^was adopted to calculate the relative expression level of the above genes. Duncan’s multiple range test program in ANOVA was used to examine the significance of the expression analysis based on the SAS 8.0 software (SAS Institute Inc., North Carolina, USA).

**Table 1:** Primers for Real-time PCR

Gene	Forward primer sequence (5΄-3΄)	Reverse primer sequence (5΄-3΄)
*MSR1*	CGATGCTCGCTCAATGACA	AATGATGGGCACGAGAACTACA
*OLR1*	CTGCTTGTCTTTGGACGCC	AGCCACGAGTAGCTGGGTT
*NFKB1*	TGGCAGCTCTTCTCAAAGCA	GACCCCTTCATCCTCTCCATC
*CD14*	TGGACCTCAGCCACAACTCG	AGCCCAGCGAACGACAAAT
*MYD88*	CGGATGGTGGTGGTTGTCT	GGAACTCTTTCTTCATTGGCTTG
*TLR4*	GGGTGCGGAATGAACTGGT	GATGATATTGGCGGCGATG
*ICAM1*	ACCCTCATCTTGGGCACTC	GGCGGCGTGGATTTCA
*β*-*actin*	AGATGTGGATCAGCAAGCAG	CCAATCTCATCTGCTTTTCTG

## Results

### Production and screening of *TLR4*-overexpressing sheep

**Fig. 1:**
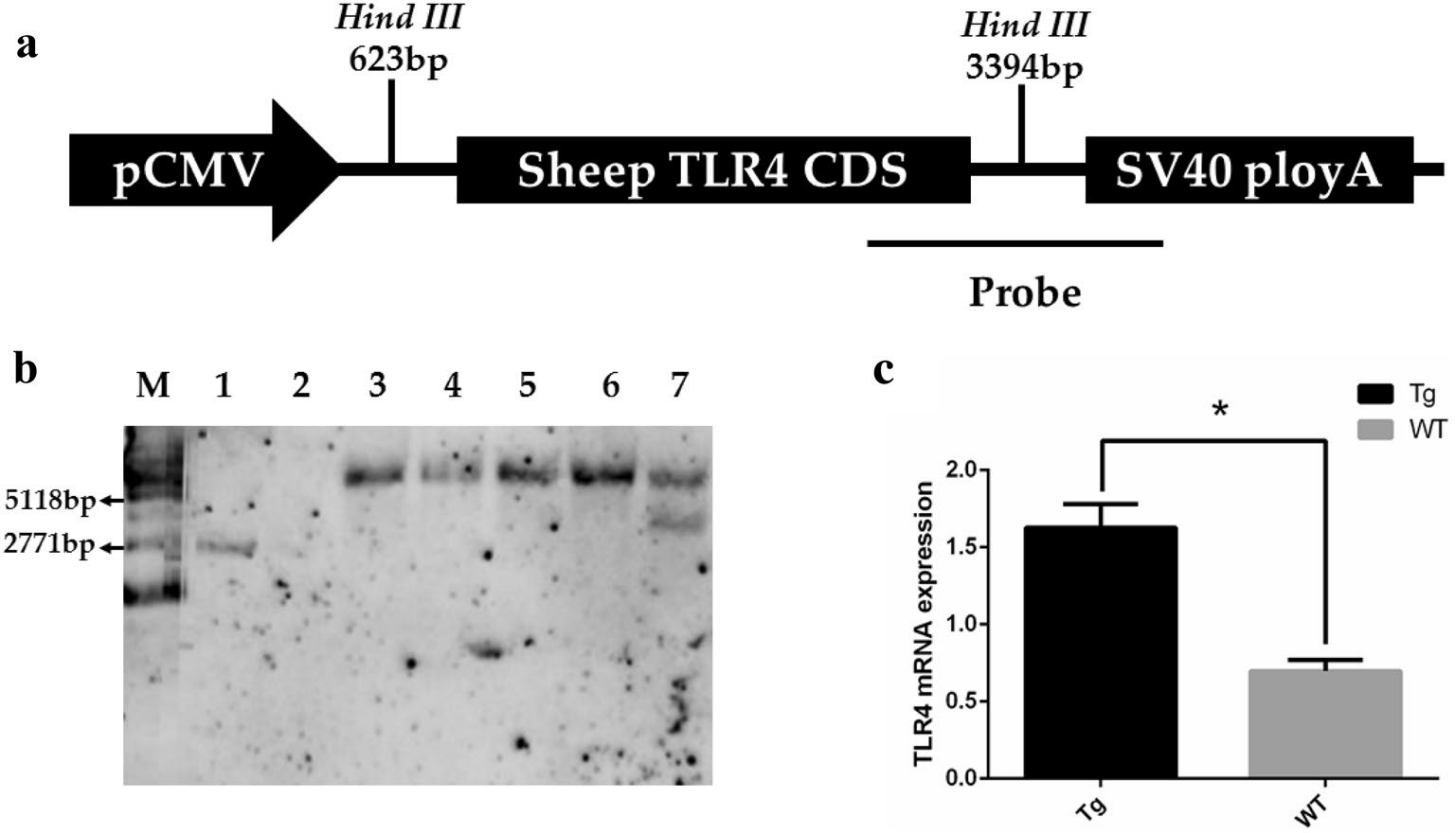
Production and screening of *TLR4*-overexpressing sheep. **a** The *TLR4* expression vector. Ovine *TLR4* CDS was inserted into the p3S-LoxP vector, which possessed the promoter CMV and SV40 polyA. The probe for Southern blot was used to screen the *TLR4*-overexpressing sheep. **b** The Southern blot analysis for the DNA sample from the *TLR4* expression vector and the ovine ear tissue. Lane 1 is 5 × sample of the *TLR4* expression vector, Lane 2 is a 1 × sample of the *TLR4* expression vector, Lanes 3–6 are WT individuals, and Lane 7 is a Tg (*TLR4*-overexpressing) individual. **c** The mRNA expression of *TLR4* in peripheral blood mononuclear cells quantified with real-time PCR

The CDS of *TLR4* from ovine spleen was inserted into the p3S-LoxP vector, which possess the promoter CMV and SV40 polyA (Fig. 1a). As shown in Fig. 1b (using the southern hybridization probe), the expected size of the exogenous *TLR4* fragment is 2771 bp, and that of the endogenous *TLR4* fragment is 5118 bp. The expression of *TLR4* for PBMC was higher in Tg sheep than in WT sheep (*p *< 0.05; Fig. 1c). These results indicated that *TLR4*-overexpressing sheep were successfully generated.

### Overview of RNA-sequencing data

In order to evaluate the gene expression of *TLR4*-overexpressing sheep, and study the co-expressed and downstream genes for *TLR4*, the PBMC derived from *TLR4*-overexpressing (Tg) and wild type (WT) sheep were chosen as the research object. Three biological repetitions of the two groups (Tg and WT) were selected to construct the libraries for sequencing. An average total of 89,826,127 raw reads were obtained. After discarding low-quality reads, contaminants and adapter–adapter ligation reads, an average total of 89,361,187 clean reads were obtained from each library.

The Q20 and Q30 indexes for the six libraries were all higher than 97% and 95%, respectively. This result confirms that the quality of the sequencing data is high, and that the filtered libraries could be used for follow-up statistical analyses (Additional file 1). As shown in Additional file 2, the clean reads were assembled and mapped to the ovine genome. The percentages of the total mapped and uniquely mapped reads for the six libraries were greater than 82% and 76%, respectively, while the multiple mapped reads accounted for less than 6%. The clean reads matched the positive and negative strands of the ovine genome and accounted for the same percentage in all six libraries. All of the mapped indexes in Additional file 2 showed no significant differences between the Tg and WT group. The expression level of a read count greater than 1 was defined as a detectable gene. An average of 18,754 and 19,530 known genes were identified in the Tg and WT libraries, respectively (Additional file 3). There was no significant difference in the amount of gene expression between the Tg and WT libraries.

**Fig. 2:**
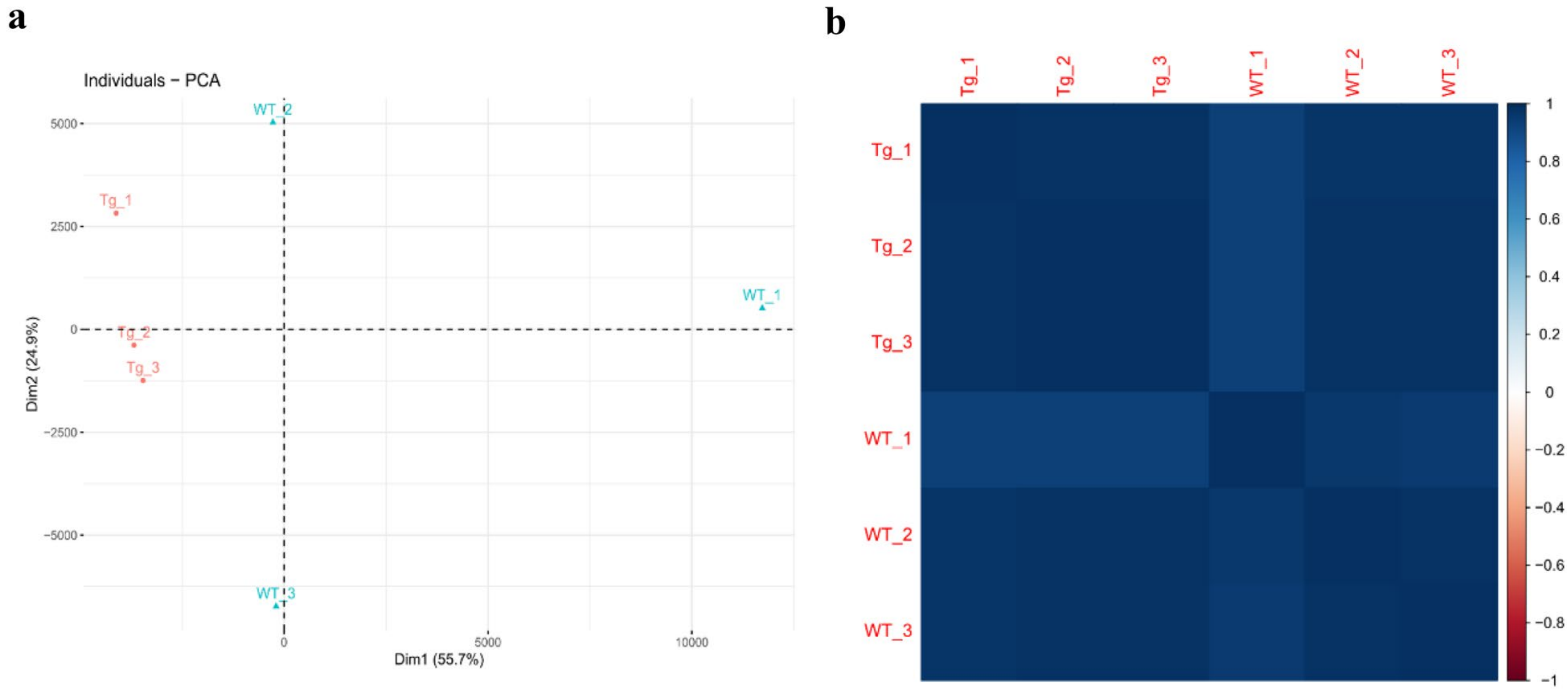
Sample correlation analysis using PCA and Pearson correlation. **a** PCA analysis of the gene expression level for peripheral blood mononuclear cells. **b** Pearson correlation analysis of the gene expression level for peripheral blood mononuclear cells

As shown in Fig. 2a, PCA revealed that the Tg samples formed a lesser variable population compared to WT, although the WT sheep mildly deviated from the other individuals. However, the Pearson correlation coefficients for each experimental individual were all higher than 0.93, which indicates that the two experimental groups possess few differences in their PBMC gene expression (Fig. 2b). This suggests that the experiments on transgenic vector design and pronuclear microinjection are reliable for *TLR4* overexpression, which did not lead to the excessive disordered expression of endogenous genes.

### Analysis of differentially expressed genes between Tg and WT groups

**Fig. 3:**
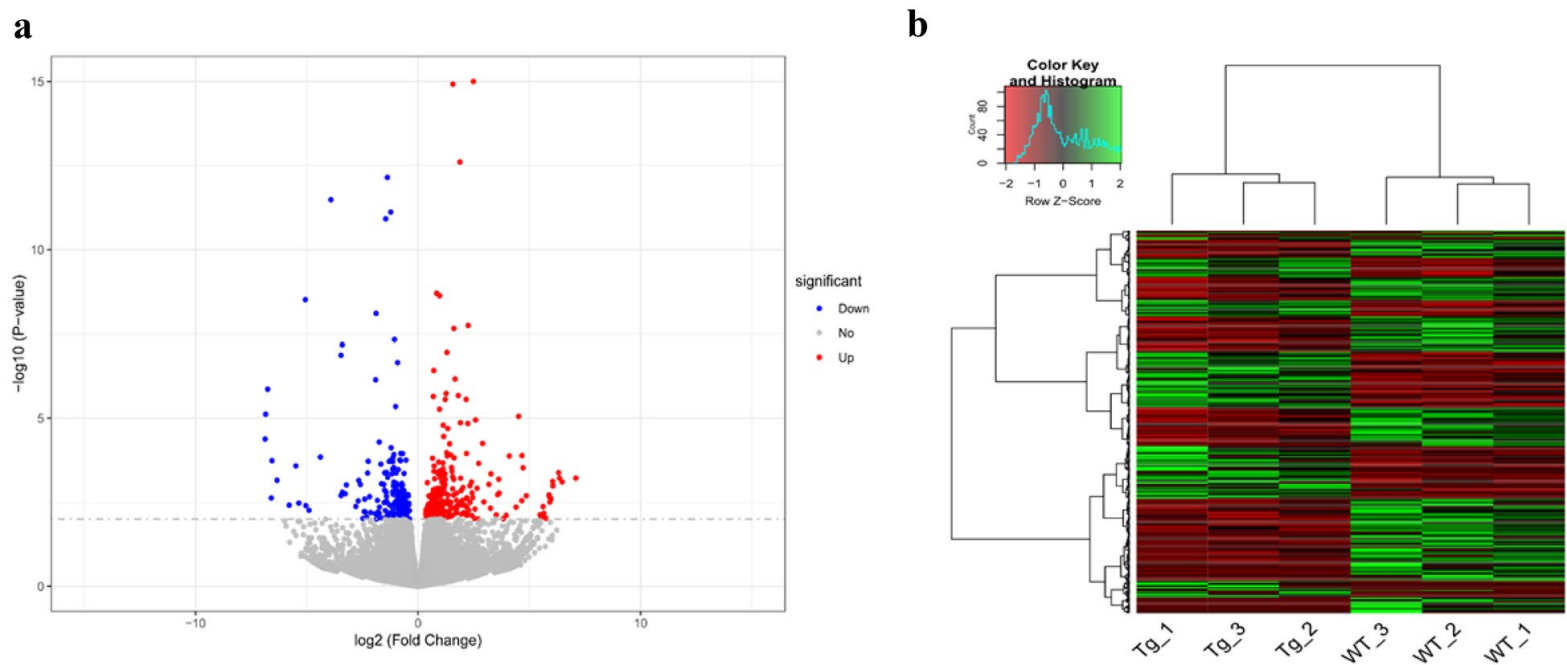
Analysis of differentially expressed genes. **a** Volcano plot for screening DEGs (*p* < 0.01). **b** Hierarchical cluster analysis of DEGs (*p* < 0.01)

Considering the need to screen a sufficient amount of DEGs for subsequent GO and KEGG pathway analysis, the uncorrected *p*-value of < 0.01 was adopted as the threshold for DEGs screening. As shown in Additional file 3, we found that a total of 338 known genes and 85 novel transcripts were differentially expressed between Tg and WT libraries (*p* < 0.01). Compared to the WT libraries (*p* < 0.01), there were 150 vs 188 known genes and 30 vs 55 novel transcripts were up-regulated vs down-regulated in the Tg libraries (Fig. 3a). For the above DEGs, a hierarchical cluster diagram was drawn to present the genes clustered together according to their similar expression patterns. All of the *TLR4*-overexpressing individuals were clustered together from WT individuals (Fig. 3b). Immunoglobulin related and some internalization-associated genes were differently expressed between Tg and WT groups (Table 2).

**Table 2:** Immunoglobulin and internalization-associated genes expression in ovine Peripheral Blood Mononuclear Cells

gene_id	AVER-read-Tg	AVER-read-WT	*p*-value	gene_name	gene_description
ENSOARG00000009395	1876.67	769	1.02 × 10^−12^	–	Immunoglobulin C1-set domain
ENSOARG00000014841	453.33	126.67	3.47 × 10^−7^	–	interleukin-8 like
ENSOARG00000012641	1340	819	0.000165	–	Immunoglobulin V-set domain
ENSOARG00000021012	825	412.67	0.001029	OLR1	Lectin C-type domain
ENSOARG00000025180	1908.33	1257.33	0.002252	CD14	–
ENSOARG00000005792	1937.67	1275.33	0.002533	TLR4	TIR domain
ENSOARG00000001031	944	874.67	0.280697	MYD88	TIR domain
ENSOARG00000009626	374.33	329	0.466921	MSR1	Macrophage scavenger receptor triple helix repeat
ENSOARG00000012718	5411	5626.67	0.79464	NFKB1	Death domain|
ENSOARG00000015656	140	156.33	0.98454	ICAM1	Intercellular adhesion molecule

gene_id indicates ensemble gene Id, AVER-read-Tg indicates average read counts in Tg group, AVER-read-WT indicates average read counts in WT group

### GO enrichment and KEGG pathway analysis of DEGs

**Fig. 4:**
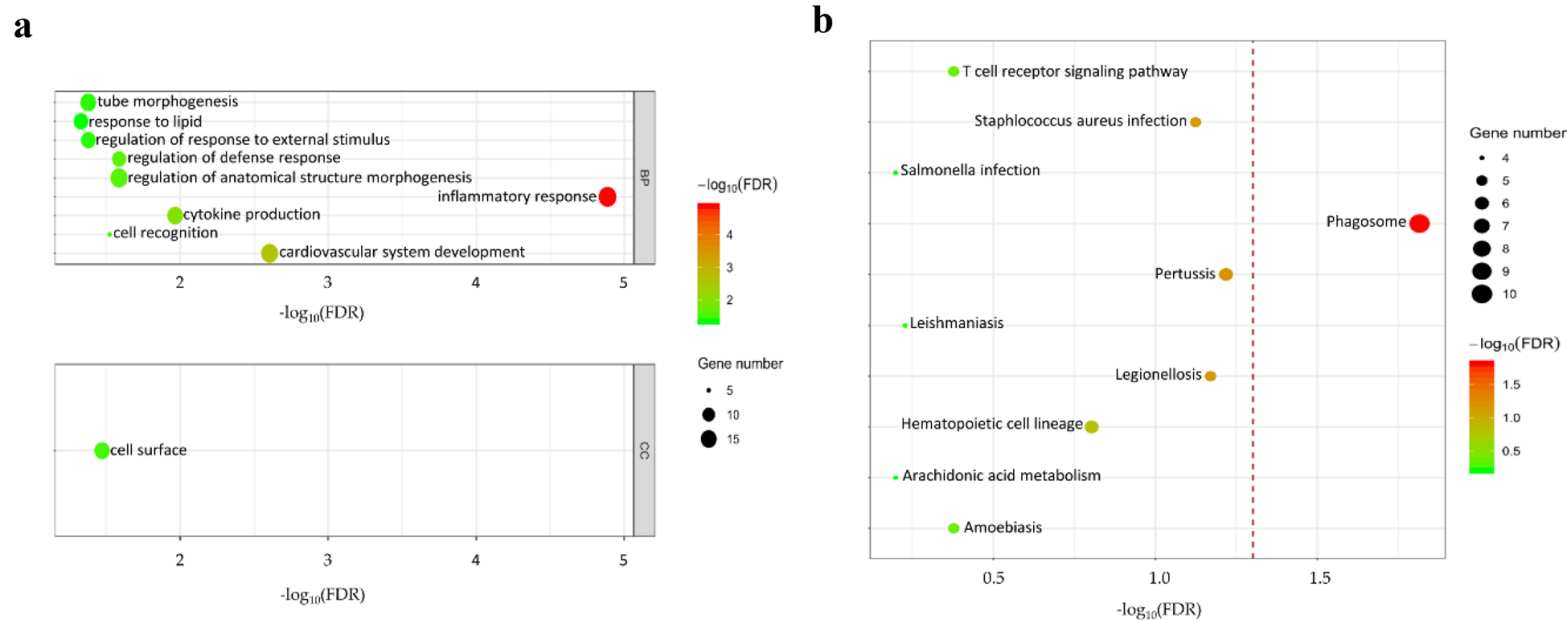
GO and KEGG pathway enrichment analysis of differentially expressed genes. **a** GO enrichment analysis of DEGs. 9 and 1 significant GO terms with an FDR significance level < 0.05, for BP and CC ontologies, respectively, are presented in the figure. **b** KEGG pathway analysis of DEGs. Under a significance level of FDR < 0.05, only the pathway of Phagosome (to the right of the dotted line) was significantly enriched

The above DEGs were mapped to the bovine GO database, and 226 genes were unambiguously mapped to unique Entrez gene IDs. Moreover, 123, 89 and 113 genes were separately annotated to the ontologies of biological process (BPs), cellular component (CCs) and molecular function (MFs), respectively. In the reference list of 23,134 Entrez gene IDs, 9011, 6224 and 7960 genes were annotated to the mentioned three ontologies. Compared with the reference background gene, 9 and 1 GO terms were significantly (FDR < 0.05) enriched, respectively, in the BP and CC ontologies (Additional file 4). Notably, the GO terms for inflammatory response, cell recognition, regulation of defense response, and regulation of response to external stimulus were significantly (FDR < 0.05) enriched in the BP ontology, while the GO term of cell surface was significantly (FDR < 0.05) enriched in CC ontology (Fig. 4a).

The KEGG pathway analysis was also performed based on the DEGs, of which 226 genes were unambiguously mapped to unique Entrez gene IDs, and 102 genes were annotated to the bovine KEGG database (Additional file 5). Among the reference list of 23,134 Entrez gene IDs, 8165 IDs were annotated to the pathways of KEGG. Compared with the reference background gene, only the pathway of the Phagosome was significantly (FDR < 0.05) enriched (Fig. 4b). This significantly enriched pathway coincides with the results of *TLR4*-overexpressing sheep, which exhibited a more sensitive response to Gram-negative bacteria.

### Analysis of genes associated with the expression pattern of *TLR4* in Tg sheep

**Fig. 5:**
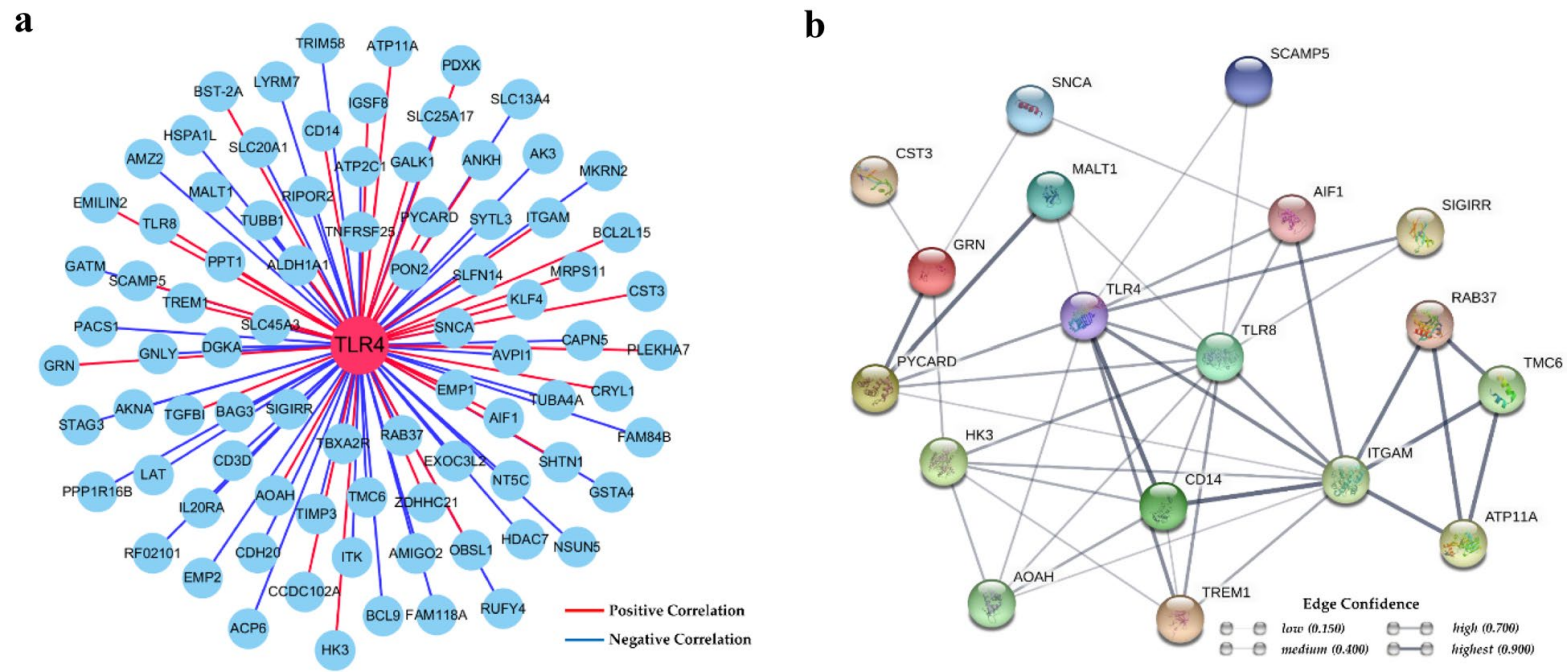
Analysis of the genes associated with the expression pattern of *TLR4*. **a** The mRNAs of 84 known genes that are strongly associated with the expression pattern of the *TLR4* gene. The red line represents a positive correlation, and the blue line represents a negative correlation. **b** The interaction network of 18 proteins backed up by the human database in String

The main purpose of our previous experiment was to increase the expression level of the *TLR4* gene in sheep to enhance their resistance to Gram-negative bacteria [10, 13]. However, gene expression is a complex interaction system. To investigate whether overexpression of *TLR4* affects the expression of other genes in sheep, a Pearson correlation analysis were performed on all of detected genes in Tg sheep (Additional file 6, Table 1). Then, all known genes whose expression pattern significantly (*p* < 0.05) associated with *TLR4* were presented in Table 2 of Additional file 6. A total of 84 known genes were strongly associated with the expression patterns of the *TLR4* gene (*p* < 0.05); the positive and negative correlation relationships are shown in Fig. 5a. Based on the human database in String (https://string-db.org/), some nodes in these 84 known genes without data support were removed. An interaction network of the remaining 18 proteins is provided in Fig. 5b, which shows that *TLR8*, *CD14*, and *ITGAM* are the important genes for the biological functions of *TLR4*.

### Real-time PCR of internalization-associated genes

**Fig. 6:**
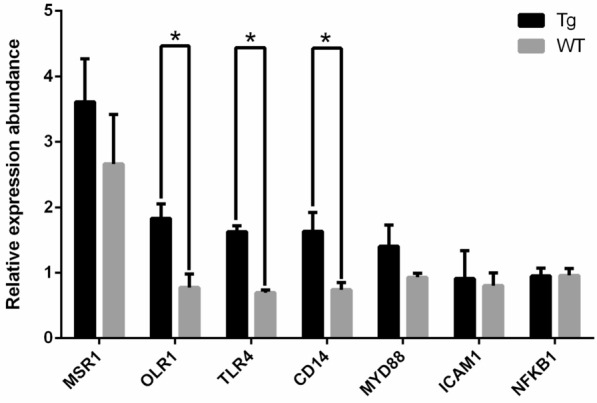
Real-time PCR of internalization-associated genes. Symbol of “*” indicate significant differences (*p* < 0.05) between the Tg and WT groups

Seven genes (*MSR1*, *OLR1*, *NFKB1*, *CD14*, *MYD88*, *TLR4*, and *ICAM1*) involved in the process of bacterial internalization were selected to verify their expression levels in the peripheral blood mononuclear cells between the Tg and WT groups. Gene expression values from separate samples detected by real-time PCR and RNA-Seq were subjected with Pearson correlation analysis in Additional file 7. The correlation coefficients between the two methods in the seven genes expression detection ranged from 0.697 to 0.958 which means that the seven genes expression levels detected by real-time PCR verified the reliability of RNA-Seq in gene expression detection. Both of the two methods showed that the mRNA expression levels of *OLR1, CD14,* and *TLR4* were significantly different between Tg and WT groups (*p* < 0.05; Fig. 6; Additional file 3).

## Discussion

Creating genetically modified organism (GMO) may be an important approach for humans to tackle future challenges in food crisis. To date, GM technology has been widely used in crops for insect-resistance or herbicide tolerance such as in maize, soybean, cotton, and canola [22]. The production of GM animals has been envisioned as an alternative to boost food quality, animal yield, and for the production of bioproducts that can be used for the benefit of the human and animal population [23]. Strict supervision with biotechnology should be applied in the GM industry, as such supervision will be favourable for the industry’s healthy development. Comparative transcriptional and protein-expression based on single gene or high-throughput approaches have been adopted to evaluate the existence of unintended modifications [24–26]. In the present study, the differential expression profiles between *TLR4*-overexpressed and wild type sheep were explored by RNA-sequencing, the results of which may help evaluate the safety of GM sheep, and analyse some specific genes whose expression patterns are associated with *TLR4*.

Brucellosis which caused by *Brucella melitensis* (Gram-negative bacteria) infection has been eradicated from cattle in some developed countries, while it remains a ‘multiple burdens’ disease causing losses in the animal husbandry economy in developing countries [27–29]. Vaccination and slaughter are the basic approaches used for controlling and eradicating brucellosis. The above approaches not only have the risks of biosecurity and economic loss, but have also failed in southern Europe [30–32]. Fortunately, genetic modification for animal disease-resistant breeding is gradually being achieved. Some studies have shown that genetic modification technology can be successfully applied for the prevention and control of diseases in chickens, cattle, and other animals [33–35]. In previous work, GM sheep with *TLR4* overexpression were demonstrated to be effective against Gram-negative bacteria, and the growth performance, reproductive traits and offspring survivability of such sheep have also been researched and evaluated [7, 9, 10, 36].

In the present study, Southern blot and real-time PCR were performed to verify the successful generating of *TLR4*-overexpressing sheep. In our previous study, PBMC collected from the same sheep were cultured for generating macrophages, then these macrophages from the Tg group presented a significantly higher expression of TLR4 under the analysis of flow cytometry and Western blot [37]. Pronuclear microinjection technology may cause the risks of uncertainty in gene integration sites, and make endogenous genes expression disordered. As a robust and cost-efficient approach, RNA-Sequencing became a good method to identify and quantify genes expression in whole genome-wide. Since *TLR4* mainly plays a biological role in immune cells, PBMC were selected as the sequencing objects. A total of 402 zygotes were microinjected with *TLR4* overexpression vectors, and only three male and four females were found to be positive for carrying exogenous *TLR4*. The low number of sample diminishes the reliability of these statistics. PCA and Pearson correlation analyses failed to distinguish the two groups. At the current statistical level, we found little difference in the overall gene expression levels between the Tg and WT libraries. The results of these analyses demonstrate that previous research of transgenic vector design and pronuclear microinjection are reliable for *TLR4* overexpression [7, 9, 10, 36].

Recently, the pronuclear microinjection technique was also applied to produce sheep with β-Catenin-overexpression; a small number of 113 DEGs were found between the transgenic sheep and the WT group using RNA-Seq [38]. In our experiment, 338 known genes were differentially expressed by screening (*p* < 0.01). When we set the significance threshold of Padj (FDR) < 0.05, only a total of 49 DEGs were screened in the transgenic sheep and WT group. The above result revealed that only a few genes have changed their expression levels in the *TLR4*-overexpressing sheep. However, these fewer DEGs are not sufficient to detect disturbances in the signal pathway, making it challenging to explain biological issues. Therefore, the lower standards (uncorrected *p* < 0.01) of DEGs screening becomes a necessary choice to detect the signal pathway interference in this study.

*TLR4* is expressed higher in Tg individuals which agrees with the screening results [37, 39]. A further analysis found that the DEGs were enriched in the GO terms of inflammatory response, cell recognition, regulation of defence response, and regulation of response to external stimulus. A high level of inflammatory response and the subsequent activation of mononuclear macrophages were necessary to identify LPS [37]. Ancestor chart for GO:0031347 shows that regulation of defense response is a regulation of response to external stimulus (https://www.ebi.ac.uk/QuickGO/term/GO:0031347). Combined with the enriched GO terms of inflammatory response and cell recognition, we speculate that the resistance to Gram-negative bacteria of PBMC in Tg sheep is superior to that in WT sheep. Furthermore, the DEGs were enriched in the only KEGG pathway of the Phagosome. These enrichment results reveal that *TLR4*-overexpression in our experiment may strengthen the innate immune response in sheep. As the first line of ovine defence against invading pathogens, the immunocytes of peripheral blood play important roles in inflammation, which is crucial for resisting Gram-negative bacteria [37, 40]. In addition, the phagocytosis of macrophages is also closely associated with the inflammatory process [41]. Many previous works have confirmed the results of the enrichment analysis in the present study [6, 7, 37, 42, 43]. To date, no information about *Ovis aries* is included in the database of WebGestalt 2019 for enrichment analysis. Therefore, we had to convert the ensemble gene id into gene symbol, then the gene symbols were uploaded as the list of interest which mapped into bovine KEGG pathway database in WebGestalt 2019. However, many DEGs have no corresponding gene symbols (or gene name), and about a third of the DEGs are lost from the initial list. The above reasons caused the very low number of genes among DEGs which could map to terms in GO and KEEG databases. We hope the ovine database for GO and KEGG pathway become more complete in the future, then we will obtain a more accurate enrichment result.

The endocytosis of TLR4 in innate immune response is the core process of defence against invasion of Gram-negative bacteria [37]. Under the stimulation of LPS, CD14 controls a microbe-specific endocytosis pathway for internalizing TLR4 signal transduction [42]. The expression level of *CD14* in the Tg group is significantly higher than the WT in peripheral blood mononuclear cells, which verifies the research of Tanimura et al. [43] showing that *CD14*-overexpression enhances TLR4 endocytosis in the B cell line. In the present study, only *TLR4* and *CD14* were up-regulated, while the downstream genes in the pathway of NF-κB have no difference in the expression level. In combination with our previous research [7], we can infer that the higher resistance to Gram-negative bacteria may be presented when stimulated by LPS. Cervantes et al. [44] had demonstrated that human TLR8 is able to sense bacterial RNA released within phagosomal vacuoles, and maintains necessary crosstalk with other endosomal TLRs. ITGAM (also known as CD11b), which is expressed in macrophages, plays a critical role in pathogen recognition and phagocytosis [45]. In the present study, the expression patterns of *CD14*, *TLR8*, and *ITGAM* were strongly associated with *TLR4*, and the above three genes are an intermediary for the other significantly associated genes. Therefore, *CD14*, *TLR8*, and *ITGAM* are considered to be involved in the immune response of *TLR4*-overexpressing sheep.

## Conclusions

In summary, the PCA and Pearson correlation analyses indicated that the overall gene expression patterns of the PBMC in Tg and WT sheep are similar. Moreover, the DEGs (*p* < 0.01) between the Tg and WT libraries were significantly enriched in GO in terms of inflammatory response, cell recognition, regulation of defense response, and regulation of response to external stimulus (FDR < 0.05); they were especially enriched in the sole KEGG pathway of the Phagosome (FDR < 0.05). The above results reveal that overexpressed *TLR4* in our experiment strengthened the ovine innate immune response by increasing the phagocytosis in PBMC.

## Supplementary information

**Additional file 1.** Statistics of sequencing quality.

**Additional file 2.** The distribution of the sequenced mRNA.

**Additional file 3.** Differential expression of the mRNA in ovine Peripheral Blood Mononuclear Cells for Tg and WT individuals.

**Additional file 4.** GO enrichment analysis of DEGs.

**Additional file 5.** KEGG pathway analysis of DEGs (Top 10).

**Additional file 6.** Pearson correlation analysis of gene expression.

**Additional file 7.** Expression levels of the seven selected genes in each samples detected by real-time PCR and its correlation with RNA-seq.

## Data Availability

The datasets used and/or analyzed during the current study are available from the corresponding author on reasonable request.
